# Sulfamethoxazole – Trimethoprim represses *csgD *but maintains virulence genes at 30°C in a clinical *Escherichia coli *O157:H7 isolate

**DOI:** 10.1371/journal.pone.0196271

**Published:** 2018-05-02

**Authors:** Gaylen A. Uhlich, Elisa Andreozzi, Bryan J. Cottrell, Erin R. Reichenberger, Xinmin Zhang, George C. Paoli

**Affiliations:** 1 Molecular Characterization of Foodborne Pathogens Research Unit, Eastern Regional Research Center, Agricultural Research Service, United States Department of Agriculture, Wyndmoor, Pennsylvania, United States of America; 2 BioInfoRx, Inc, Madison, Wisconsin, United States of America; New York State Department of Health, UNITED STATES

## Abstract

The high frequency of prophage insertions in the *mlrA* gene of clinical serotype O157:H7 isolates renders such strains deficient in *csgD*-dependent biofilm formation but prophage induction may restore certain *mlrA* properties. In this study we used transcriptomics to study the effect of high and low sulfamethoxazole–trimethoprim (SMX-TM) concentrations on prophage induction, biofilm regulation, and virulence gene expression in strain PA20 under environmental conditions following 5-hour and 12-hour exposures in broth or on agar. SMX-TM at a sub-lethal concentration induced strong RecA expression resulting in concentration- and time-dependent major transcriptional shifts with emphasis on up-regulation of genes within horizontally-transferred chromosomal regions (HTR). Neither high or low levels of SMX-TM stimulated *csgD* expression at either time point, but both levels resulted in slight repression. Full expression of Ler-dependent genes paralleled expression of group 1 *pch* homologues in the presence of high *glrA*. Finally, *stx*_*2*_ expression, which is strongly dependent on prophage induction, was enhanced at 12 hours but repressed at five hours, in spite of early SOS initiation by the high SMX-TM concentration. Our findings indicate that, similar to host conditions, exposure to environmental conditions increased the expression of virulence genes in a clinical isolate but genes involved in the protective biofilm response were repressed.

## Introduction

Enterohemorrhagic *Escherichia coli* (EHEC) cause intestinal disease characterized by hemorrhagic colitis that can progress to the severe renal-associated sequelae, hemolytic uremic syndrome (HUS). In the United States, the highest incidence of EHEC clinical cases, large outbreaks, and HUS are associated with serotype O157:H7 [1, 2]. Serotype O157:H7 contains a number of prophage- and plasmid-encoded virulence factors of which Shiga toxins (Stx2 alone or in addition to Stx1), the locus of enterocyte effacement (LEE), and the large F-like plasmid (pO157) are the most important [3, 4]. However, multivariate analyses testing the correlation of EHEC strain and patient factors with HUS have agreed that the most important predictors of HUS are Stx2 and LEE, while the significance of pO157 is less clear [5, 6].

A remarkable feature of the sequenced *E*. *coli* serotype O157:H7 strains is the abundance of horizontally transferred foreign DNAs that are inserted in their genomes. For instance, the Sakai reference strain (accession #NC_002695.1) consists of conserved *E*. *coli* core genes along with strain-specific DNA that comprises more than a fourth of its genome [7]. Nearly half of the strain-specific DNA consists of prophage (Sp1-Sp18) or prophage-like (SpLE1-SpLE6) elements, so designated because they encode no bacteriophage genes other than integrases. Sakai foreign DNA segments specifically involved in virulence have also been assigned into six groups termed pathogenicity islands (EPAI 1–6), three of which (EPAI 3, 5 and 6) are within Sp and SpLE regions (Sp17, SpLE3, and SpLE4, respectively) [8]. In Sakai, Sp15 carries *stx*_*1*_, Sp5 carries *stx*_*2*,_ and LEE is encoded in SpLE4 [9].

LEE is essential for the formation of attaching and effacing lesions by both EHEC and enteropathogenic *E*. *coli* (EPEC) [10]. LEE contains five major polycistronic operons (LEE1-5) and several smaller bicistronic operons or individual genes [11]. LEE1-3 are involved with a type III secretion system (TTSS), LEE4 includes several secreted proteins, and LEE5 contains the genes encoding the intimin adhesin (*eae*) and its translocated host receptor, Tir (*tir)*. The first gene of the LEE1 operon (*ler*) encodes the Ler transcriptional regulator, which activates most LEE genes [12, 13]. The global regulator H-NS has a silencing effect on LEE including Ler/LEE1. This suppression can be relieved by Ler, whose amino acid sequence has similarity to H-NS in the DNA-binding domain [14]. The complex regulation of Ler has been described in detail in reviews [15–17]. The *grlA* transcription factor, which forms a small operon between LEE1 and LEE2 along with its suppressor *grlR*, is essential for full Ler/LEE activation and is reinforced by Ler in a positive feedback loop [18, 19]. Both *ler* and *grlRA* are subject to quorum sensing regulation mediated by host epinephrine and norepinephrine [20, 21]. Ler is also activated in both EPEC and EHEC by transcriptional regulator, PerC [22, 23]. In EPEC, a single PerC activates LEE. However, in EHEC Ler is controlled by the combined activities of different *perC* homologues (*pch*) encoded within various Sp or SpLE [24]. Five such genes encoded within Sakai can be assigned into three groups based on DNA similarity, size, and functionality: group1 (*pchA*, *pchB*, *pchC*), group 2 (*pchD*) and group 3 (*pchE*). Various other transcription factors such as Fis, IHF, BipA, Hha, GrvA, and the products of genes from a nonfunctional TTSS, *etrA* and *eivF*, add to the control of LEE [15–17, 25].

Because of deletions, insertions, and Insertional Sequence (IS) elements in prophage essential regions, Sp and SpLE were considered degenerate with questionable capacity for excision, packaging, and lateral transmission [7]. However, Asadulghani *et al*. [26] showed that nine of the 18 prophages can excise from the Sakai chromosome and replicate. Both Sp5 (*stx*_*2*_) and Sp15 (*stx*_*1*_) excised and replicated following SOS induction by mitomycin C (MMC); however, LEE, encoded in SpLE4, did not [26]. Lambda prophage induction is triggered by the SOS response, secondary to agents or events initiating cell cycle arrest and/or DNA repair [27]. Exposed single-stranded DNA activates RecA, a member of the SOS regulon, which assists autocatalytic inactivation of the SOS regulon suppressor, LexA. The lambda prophage repressor cI becomes susceptible to RecA cleavage during the SOS response and the lytic cycle is de-repressed. DNA damaging antibiotics, including quinolones and sulfamethoxazole-trimethoprim (SMX-TM) have been shown to induce the SOS response and elevate *recA* expression [28, 29]. For instance, trimethoprim, which reduces the cellular pool of tetrahydrofolate required for purine synthesis, induces DNA damage prior to its bacteriocidal effects and was shown to strongly induce the SOS regulon [30]. A consequence of antibiotic treatment of Shiga toxin-producing *E*. *coli* (STEC) patients is the potential for precipitation or enhancement of HUS [28]. Host cell damage caused by Shiga toxin is central to STEC HUS and prophage induction results in dramatic increases in Stx production [1, 31]. Although a definitive relationship between antibiotic treatment and HUS has been difficult to confirm, it is regarded as a best practice to avoid administration of antibiotics during treatment of STEC infections [32].

In addition to carrying toxin genes (*e*.*g*. *stx*) and regulatory factors (*ler*, *grl*, *perC*, etc.), Sp and SpLE encode effector proteins, which are injected through the LEE TTSS. Tobe *et al*. [33] confirmed 39 functional effector proteins encoded on exchangeable effector loci in strain O157:H7 Sakai.

Insertion of foreign DNA or induction of integrated genetic elements can also influence the expression of the genes surrounding the bacteriophage insertion site. For example, run off replication during phage induction can amplify large numbers of flanking genes [34]. Prophage insertion also has a profound effect on O157:H7 biofilm-forming properties as a result of disruption of *mlrA*, the gene carrying the insertion target for the prophage that often encodes *stx*_*1*_ [35]. The *mlrA*-encoded transcription factor enhances RpoS-dependent expression of the essential biofilm regulator CsgD, required for complete expression of curli fimbriae and genes for cellulose biosynthesis [36]. Greater than 95% of clinical O157:H7 isolates carry a prophage in *mlrA* that disrupts the MlrA DNA-binding motif, which results in the loss or reduction of curli expression, biofilm formation, and curli-dependent Congo red (CR) dye binding [35, 37]. Spontaneous prophage excisions from *mlrA* restore a small percentage of PCR-detectable unoccupied insertion sites within growing O157:H7 populations at either host (37°C) or environmental (30°C) temperatures, and prophage-inducing agents such as SMX-TM can increase the level of cells with restored *mlrA* [35, 37]. However, plating of the induced or un-induced populations rarely identifies individuals that have lost the prophage, suggesting that prophage excisions are transient or detrimental [38]. It has also been shown that the *mlrA* portion distal to the prophage insertion site encodes truncated *mlrA* proteins that could be driven by run-off transcription [38]. When expressed as recombinant forms, these proteins restored some CR affinity, but not biofilm formation. Loss of the promoter binding region in these modified factors makes it unlikely that they function as DNA-binding transcription factors similar to wild-type MlrA. In addition to MlrA, a large network of regulators controls *csgD* expression including transcription factors, signaling molecules such as c-di-GMP, and various sRNAs [39–44]. *csgD*-dependent phenotypes can also be restored by various mutations in regulators such as the *csgD* suppressor, *rcsB* [45].

We previously showed that *E*. *coli* serotype O157:H7 clinical isolate PA20 carried a *stx*_1_ prophage in *mlrA* but that curli/biofilm production might be influenced by prophage activation and excision [35, 38]. In this study, we used transcriptomics to study the effect of sub-lethal SMX-TM concentrations on prophage induction, biofilm regulation, and virulence gene expression under stress conditions. Our results indicate that following strong RecA induction at 30°C, PA20 undergoes major transcriptional shifts with emphasis on up-regulation of genes (many virulence related) within Sp/SpLE regions and that the *csgD-*dependent biofilm regulation is repressed.

## Results

### SMX-TM effects on the PA20 transcriptome are time and concentration dependent

Although utilized for clinical therapy, SMX-TM can also affect the stability of the prophage inserted in *mlrA*, a transcription factor required for complete RpoS-dependent expression of curli fimbriae at ambient temperatures. To assess the effect of sub-lethal SMX-TM concentrations in both biofilm and virulence gene expression under environmental conditions, we compared the transcriptome of clinical isolate PA20 with and without exposure to two concentrations at two different time points using RNA-Seq (S2 Table). The RNA-Seq raw reads were submitted to the NCBI Gene Expression Omnibus (GEO) database (accession number GSE110255). A concentration of 20 μg/L SMX and 4 μg/L TM (designated 1x in this study) was the optimum concentration for inducing the *mlrA* prophage and regenerating native *mlrA* in a previous report [37]. We tested PA20 in LB media without salt (LB-NS) containing 3-fold dilutions of an inhibitory SMX-TM concentration, 15000 μg/L SMX and 3000 μg/L TM. There was a marked reduction (39%) in strain survival at concentrations greater than 555 μg/L SMX /111 μg/L TM (results not shown). That being nearly identical to 27-times the 1x concentration (540 μg/L SMX /108 μg/L TM; 27x), we used 27x as the high sub-lethal concentration in this study. Therefore, we compared gene expression differences of strain PA20 following five-hour and 12-hour growth at 30°C in LB-NS containing SMX-TM at 0, 1x, and 27x concentrations [sample comparisons 27vs0^5h^ (expression comparison of bacteria grown under the 27x concentration to no antibiotic at 5 hour time point), 27vs1^5h^, 1vs0^5h^, 27vs0^12h^, 27vs1^12h^, 1vs0^12h^]. Using a 2-fold threshold (-1.0≥ log_2_ fold change (FC) ≥1.0) for expression differences, the number of differentially expressed genes (DEG) ranged from greater than 1700 DEG in the 27vs0^12h^ comparison to only nine DEG at 1vs0^5h^ (Table 1). The 1x SMX-TM concentration had little effect on gene expression compared to untreated samples at either time point. However, the 27x concentration resulted in abundant expression differences when compared with either the 1x concentration or the untreated samples. There were also clear differences in numbers of DEG between the five-hour and 12-hour exposed cells with longer exposure resulting in a 2-fold to nearly 10-fold greater number of DEG, depending on the concentration/time-compared. When the expression difference threshold was increased from 2 fold to 3 fold (-1.5≥ log_2_ FC ≥1.5), the numbers of identified DEG genes decreased, with those at 27vs0^12h^ (the sample point showing the greatest numbers of DEG) reduced from >1700 to 961 (Table 1). The DEGs showing a three-fold or greater difference for each sample comparison are shown in S2 Table (sheets 3, 5, 7, 9, 11, and 13 for 1vs0^5h^, 27vs0^5h^, 27vs1^5h^, 1vs0^12h^, 27vs0^12h^, and 27vs1^12h^, respectively). Note that while the log_2_ of 3 is 1.58, for simplicity we have used 1.5 for analyses and reporting throughout this study.

**Table 1:** Total differential-expressed-genes (DEG) in RNA-Seq comparisons of serotype O157:H7 strain PA20 grown for five hours or 12 hours in LB-NS in the presence of different SMX-TM concentrations as listed.

Comparison	DEG 2-fold up Log_2_ FC ≥1	DEG 2-fold down Log_2_ FC ≤-1	DEG 3-fold up Log_2_ FC ≥1.5	DEG 3-fold down Log_2_ FC ≤-1.5
**1x vs 0**^**5h**^	0	9	0	4
**27x vs 1x**^**5h**^	387	368	146	133
**27x vs 0**^**5h**^	167	285	75	111
**1x vs 0**^**12h**^	9	14	1	7
**27x vs 1x**^**12h**^	777	589	520	244
**27x vs 0**^**12h**^	1013	718	633	328

1x, 20 μg/L SMX and 4 μg/L TM; 27x, 540 μg/L SMX and 108 μg/L TM

### Differential expression is greater in horizontally transferred regions (HTR)

The greatest numbers of total DEGs were observed in the 27vs0^12h^ and 27vs1^12h^ comparisons. Under such conditions, 961 (17.6%) and 764 (14.0%), respectively, of the 5448 total mapped genes showed a 3-fold or greater differential expression (Table 1). However, under certain sampling conditions, the percentages of DEG identified among the HTR (Sp, SpLE, EPAI, and plasmid pO157) were greater than the percentages associated with the total genome. For instance, at 27vs0^12h^ and 27vs1^12h^, 452 (36.0%) and 430 (34.2%), respectively, of the 1256 genes that mapped to HTR were differentially regulated, while the proportions of DEG of the 4192 backbone genes were only 12.1% (509) and 8.04% (337 genes), respectively. In contrast, there was little difference in the DEG proportions between HTR and the complete genomic complement at the 1vs0^12h^ and all 5-hour sampling points.

Moreover, the high proportions of DEG noted in the HTR at the 27vs0^12h^ and 27vs1^12h^ samplings were predominately the result of increased numbers of up-regulated rather than down-regulated genes. The percentage of up-regulated DEG in the HTR was 27.6 (347/1256) but the down-regulated percentage was only 8.8 (110/1256). In comparison, the total DEG percentage in the backbone genome at 27vs0^12h^ was only 6.9 (291/4192). Tested under these conditions, SMX-TM had a greater influence–predominately through activation rather than suppression–on genes residing within DNA elements of foreign origin, likely due in part to initiation of the SOS response. Indeed, differential expression of *recA* and *lexA* were each >3 fold at the 27x concentration vs. either the 1x concentration or the no antibiotic controls at both five hours and 12 hours (Table 2).

**Table 2:** Differential expression (log_2_ FC) of selected biofilm, virulence, and regulatory genes derived from RNA-Seq comparisons of serotype O157:H7 strain PA20 grown for five hours or 12 hours in LB-NS in the presence of different SMX-TM concentrations as listed. Comparisons with a log_2_ FC ≥1.5 or ≤-1.5 (fold change ≥3) are shown in bold.

Gene	1vs0^5h^	27vs1^5h^	27vs0^5h^	1vs0^12h^	27vs1^12h^	27vs0^12h^
*mlrA* distal to prophage	-0.08	-0.28	-0.36	0.38	2.83*	3.22*
*csgD*	-0.88	-0.34	-1.22*	-0.69	-0.36	-1.06*
*csgB*	-0.10	-0.42	-0.52	-1.35	0.02	-1.33
*pgaD*	0.47	-0.18	0.30	0.41	1.95*	2.36*
*csrB*	0.001	-0.80*	-0.81*	0.52	-2.19*	-1.66*
*cpxA*	-0.35	0.48	0.13	-0.54	0.80*	0.26
*cpxR*	-0.04	0.45*	0.41*	-0.28	0.61*	0.32*
*rcsB*	0.07	0.17	0.24	0.12	0.24	0.35
*fis*	-0.17	0.54	0.37	0.55	0.05	0.61*
*recA*	-0.11	1.97*	1.86*	-0.03	1.79*	1.77*
*lexA*	-0.16	2.33*	2.17*	-0.08	2.26*	2.18*
*pchA*	0.72	-1.29*	-0.57	0.51	1.62*	2.13* (-0.33)
*pchB*	0.93	-1.26*	-0.34	0.19	2.02*	2.21* (**1.74**)
*pchC*	0.93	-1.31*	-0.38	0.8	2.67*	3.47* (2.99*)
*pchD*	-0.21	-0.8	-1.01	0.57	-0.7*	-0.13
*pchE*	0.30	0.35	0.65	-0.41	-1.05*	-1.46*
*ihfA*	0.54	-0.54	0.00	-1.00	-1.16*	-1.00*
*ihfB*	0.34	-0.38	-0.05	0.2	-1.22*	-1.03*
*hns*	0.26	-2.18*	-1.92*	-0.5	-0.60*	-0.50*
*grlA*	0.43	2.77*	3.19*	1.46	2.21*	3.67*
*grlR*	0.71	2.63*	3.35*	1.21	1.93*	3.14*
*ler*	0.93	-0.12	0.81	1.20	1.52*	2.71*
*stx*_*1A*_	0.44	-1.61*	-1.17*	0.65	1.79*	2.43*
*stx*_*1B*_	0.52	-1.08*	-0.57	0.77	2.03*	2.80*
*stx*_*2A*_	0.99	-2.21*	-1.22	0.38	6.63*	7.01*
*stx*_*2B*_	1.30	-2.00*	-0.71	0.48	6.55*	7.03*

*, FDR *P* ≤0.05

*pch* values shown in parenthesis are results of SNP analysis; 1x, 20 μg/L SMX and 4 μg/L TM; 27x, 540 μg/L SMX and 108 μg/L TM

There were also clear differences in the percentages of DEG within the different HTRs. Sp5 (*stx*_*2*_), Sp15 (*stx*_*1*_), and SpLE4 (LEE) contained the highest DEG percentages (affected during any sample point) with 96%, 87%, and 70%, respectively (Table 3).

**Table 3:** The number and percentages of DEG in horizontally-transferred-regions (HTR). The differential expression was derived from RNA-Seq comparisons of serotype O157:H7 strain PA20 under each designated SMX-TM concentration (0, 1x, and 27x) and exposure-time sample point (five hours or 12 hours). Prophage excision after 12 hours of incubation at 27x antibiotic is indicated as either (+) for excision, (-) for no excision, (ND) not determined, or N/A if not applicable.

HTR	Genes (total)	% logFC ≥1.5 (of total)	# of DEGs (down-regulated, up-regulated)						ProphageExcision
			1vs0^5h^	27vs1^5h^	27vs0^5h^	1vs0^12h^	27vs1^12h^	27vs0^12h^	
Sp1	14	57% (8/14)	0; 0	0; 0	0; 0	0; 0	8; 0	8; 0	-
Sp2	17	24% (4/17)	0; 0	0; 0	0; 0	0; 0	2; 0	4; 0	-
Sp3	48	35% (17/48)	0; 0	0; 1	0; 1	0; 0	12; 0	15; 1	-
Sp4	69	33% (23/69)	0; 0	0; 5	1; 0	0; 0	14; 1	13; 1	-
Sp5 (Stx2)	93	97% (90/93)	0; 0	0; 11	0; 1	0; 0	87; 1	88; 1	+
Sp6	65	57% (37/65)	0; 0	0; 2	1; 0	0; 0	5; 21	5; 29	+
Sp7	25	48% (12/25)	0; 0	1; 0	0; 1	0; 0	0; 7	0; 9	+
Sp8	58	36% (21/58)	0; 0	3; 6	1; 5	0; 0	4; 9	5; 10	-
Sp9	63	35% (22/63)	0; 0	0; 1	1; 0	0; 0	4; 15	5; 14	+
Sp10	70	31% (22/70)	0; 0	2; 0	2; 0	0; 0	1; 13	2; 13	+
Sp11	69	54% (37/69)	0; 0	5; 2	2; 1	0; 0	17; 15	17; 11	+
Sp12	65	54% (35/65)	0; 0	0; 4	1; 1	0; 0	13; 9	12; 15	-
Sp13	27	11% (3/27)	0; 0	1; 0	0; 0	0; 0	0; 0	1; 1	+
Sp14	60	43% (26/60)	0; 0	0; 0	0; 1	0; 0	20; 1	23; 2	-
Sp15 (Stx1)	71	92% (65/71)	0; 0	0; 3	2; 0	0; 0	61; 0	60; 0	+
Sp16	9	11% (1/9)	0; 0	0; 0	0; 0	0; 0	1; 0	0; 0	-
Sp17/EPAI3	29	34% (10/29)	0; 0	1; 0	0; 0	0; 0	6; 1	8; 1	-
Sp18	57	14% (8/57)	0; 0	0; 3	0; 1	0; 0	4; 0	2; 0	ND
SpLE1	99	22% (22/99)	0; 0	4; 2	2; 1	0; 0	14; 2	10; 2	ND
SpLE2	16	6% (1/16)	0; 0	0; 0	0; 0	0; 0	1; 0	0; 0	ND
SpLE3/EPAI5	22	9% (2/22)	0; 0	0; 0	0; 0	0; 0	2; 0	2; 0	ND
SpLE4/EPAI6 (LEE)	56	69% (39/56)	0; 0	6; 0	6; 0	0; 0	23; 0	36; 0	-
SpLE5	12	8% (1/12)	0; 0	0; 0	0; 0	0; 0	0; 0	1; 0	ND
SpLE6	16	0% (0/16)	0; 0	0; 0	0; 0	0; 0	0; 0	0; 0	ND
EPAI1	6	33% (2/6)	0; 0	0; 0	0; 2	0; 0	0; 0	0; 0	N/A
EPAI2	14	0% (0/14)	0; 0	0; 0	0; 0	0; 0	1; 0	0; 0	N/A
EPAI4	18	44% (8/18)	0; 0	0; 0	0; 0	0; 0	4; 0	8; 0	N/A
pO157	88	50% (44/88)	0; 0	6; 0	6; 0	0; 0	31; 0	22; 0	N/A

1x, 20 μg/L SMX and 4 μg/L TM; 27x, 540 μg/L SMX and 108 μg/L TM

The low 1x SMX-TM concentration generated no differential gene expression (≥3 fold) in the HTR but there were numerous genes with 2-fold changes among the 12-hour samples indicating that the 1x concentration had only minor effects in the HTR (Table 3).

### Prophage excision and replication contribute to but are not completely responsible for differential HTR gene expression

**Fig 1:**
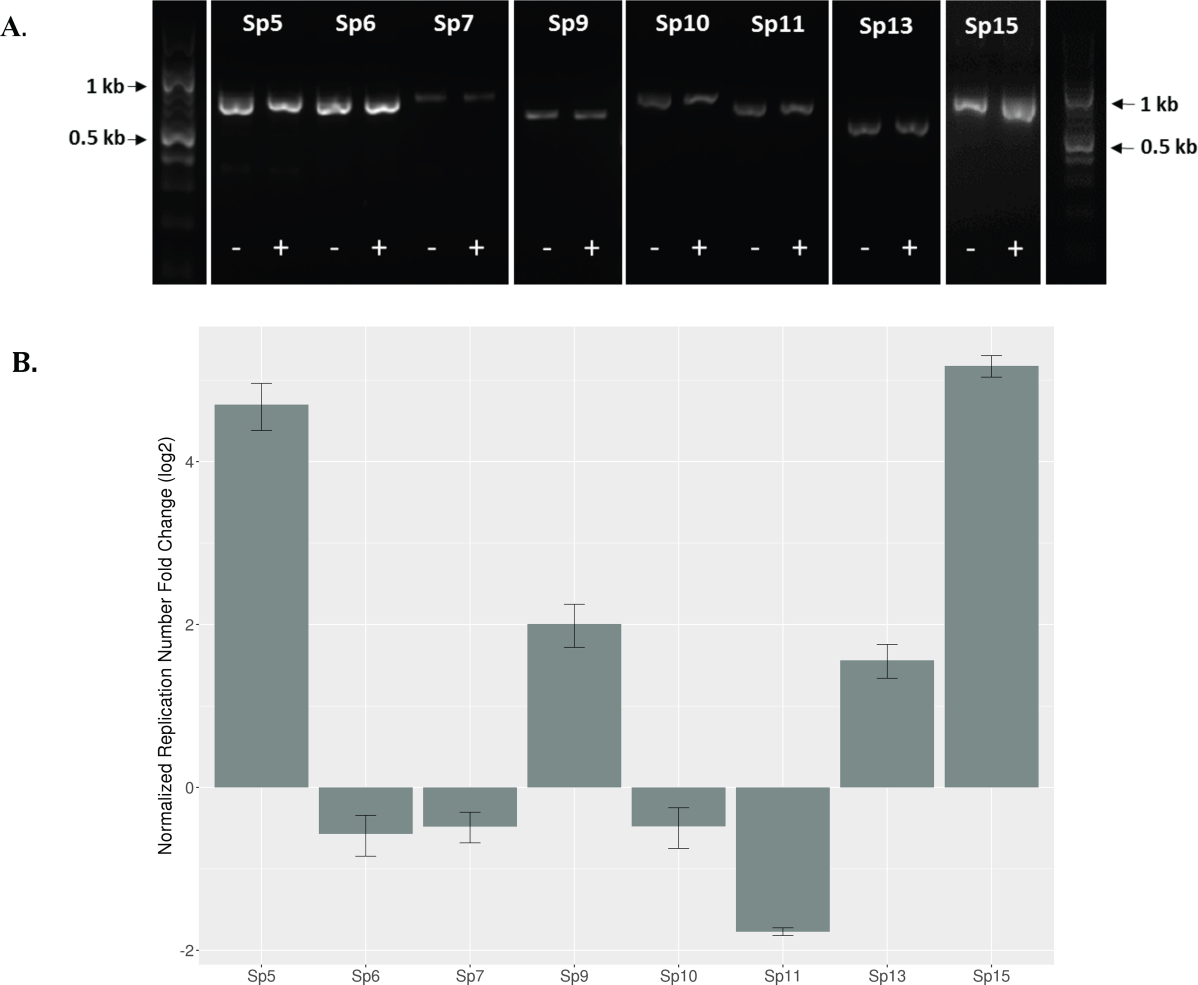
SMX-TM induced excision and fold-change estimates of PA20 Sp regions. (A) PCR amplification of the attachment site of excised and circularized Sp regions from PA20 grown on T-agar plates without (-) and with (+) an antibiotic cocktail at concentration of 27x (540 μg/L SMX and 108 μg/L TM). Sp regions 1, 2, 3, 4, 8, 12, 14, 16, 17 and SpLE4 (LEE) that did not prime amplification are not shown. Mu-like prophage of region Sp18 and SpLE except for SpLE4 were not tested. (B) Log_2_ FC copy-number differences between SMX-TM-induced and untreated (spontaneously induced) forms of the different excised and circularized PA20 SP regions. qRT-PCR was performed on DNA derived from three independent samples of PA20 grown on T-agar containing 540 μg/L SMX and 108 μg/L TM (27x), and analyzed using the 2^-ΔΔCT^method [60].

Increased copy number of bacteriophage following induction, regeneration, and bacteriophage amplification could contribute to transcript increases and favor up-regulation rather than down-regulation of encoded genes. In a study of strain Sakai, both spontaneous and mitomycin C (MMC)-induced excision and circularization were demonstrated in Sp5, Sp6, Sp7, Sp9, Sp10, Sp13, and Sp15 [26]. Sp4 and Sp14 excised only when induced by MMC and none of the highly degenerate prophage-like regions were capable of excision. When we tested DNA extracted from PA20, with or without 12-hour exposure to 27x SMX-TM, Sps5, 6, 7, 9, 10, 11, 13, and 15 all yielded PCR amplification products spanning regenerated attachment sites indicating excision and circularization (Fig 1A; Primers as listed in [26] and in S1 Table). Sp7 generated lower amounts of product but all of the eight Sps that circularized did so in both the SMX-TM treated and untreated samples. Sp18, a Mu-like prophage that does not circularize, was not tested [26]. Unlike Sakai, circularized forms of Sp4 and Sp14 were not detected in PA20. Though an unambiguous assembly was not possible, the recently published complete genome sequence of strain PA20 [46] predicts that a large chromosomal inversion had occurred between prophage elements Sp4 and Sp14. Such an inversion would miss-pair the Att sites on opposing ends of the chimeric Sp4 and Sp14 and prevent circularization, consistent with the results here. Interestingly, a second inversion with termini in Sp9 and Sp12 was also predicted in that study [46] but our finding of circularized forms of Sp9 here refutes that conclusion. There was also no product generated in our study by outward facing primers located on the ends of SpLE4, indicating no excision and amplification of the LEE EPA1 (primers in S1 Table; results not shown).

The quantitative effects of SMX-TM on prophage excisions were determined by qRT-PCR comparing induced (27x SMX-TM for 12 hours) and non-induced (spontaneously excised) samples (Primers listed in [26] and in S1 Table). SMX-TM greatly increased (log_2_ FC >4) the excised forms of Sp5 (*stx*_*2*_) and Sp15 (*stx*_*1*_), and induced smaller (log_2_ FC <2) increases in Sps 9 and 13 excisions. We also observed modest decreases (log_2_ FC >-2) in the copy number of excised Sps 6, 7, 10, and 11 following SMX-TM exposure. Although induction slightly increased the excised concentration of Sp9, translocated proteins encoded on Sp9 were strongly down-regulated by 27x SMX-TM treatment (Fig 1B).

### Differential expression of biofilm genes during SMX-TM exposure

Differential expression of *mlrA* and the *csg* genes are shown in Table 2. The only expression difference that exceeded the 3-fold change threshold was from the distal portion of *mlrA* flanking Sp15 under sample conditions 27vs0^12h^ and 27vs1^12h^. The genes from the two *csg* operons, central biofilm regulator *csgD* and curli structural protein *csgB*, showed small expression decreases at nearly every sample point, indicating a suppressive effect for SMX-TM on curli-dependent stages of biofilm development. There was little change in the expression of most other *csgD*/*B* regulators, including suppressors *cpxR*, *rcsB*, and *fis* (Table 2 and S2 Table). However, there was >3-fold higher expression of the N-acetyl glucosamine regulator *pgaD* and >3-fold lower expression of the *pgaABCD* suppressor, carbon source regulator *csrB*, generated at the 27x SMX-TM concentration at 12 hours. Such a regulatory pattern would promote biofilm maturation [47].

### SMX-TM induces time-dependent expression of virulence genes

Treatment with 1x SMX-TM did not have a significant effect on LEE gene expression but there were major expression shifts (all increases) induced by the 27x concentration compared to 1x or untreated controls (Table 4). More than 75% of the LEE genes were affected in the 27vs0 comparison at 12 hours but only 6/41 genes were affected at five hours, indicating a major dependence on exposure time. Interestingly, two of six genes enhanced at five hours were in the *grl* operon (Table 2), a major LEE regulator through its effects on *ler*. However, *ler* was not strongly induced until 12 hours. The PerC homologues (PchA, B, and C), a second major *ler* regulatory factor, were not induced by 27x SMX-TM until 12 hours. This pattern suggests that the *grlAR* genes may prime early LEE expression but full expression, dependent on the combined effects of Pchs, is delayed.

**Table 4:** Differential expression (log_2_ FC) of LEE genes derived from RNA-Seq comparisons of serotype O157:H7 strain PA20 grown for five hours or 12 hours in LB-NS at 30°C in the presence of different SMX-TM concentrations as shown. Comparisons with a log_2_ FC ≥1.5 or ≤-1.5 (FC ≥3) are shown in bold.

Locus tag	gene	1vs0^5h^	27vs1^5h^	27vs0^5h^	1vs0^12h^	27vs1^12h^	27vs0^12h^
ECs4550	*espF*	-1.34	0.57	-0.77	-0.86	2.82*	1.96*
ECs4551	*orf29*	-0.58	0.60	0.02	0.87	1.89*	2.76*
ECs4552	*escF*	-0.34	0.10	-0.24	1.26	1.96*	3.23*
ECs4553	*cesD2*	0.16	-0.39	-0.23	-0.16	0.69*	0.53*
ECs4554	*espB*	-0.41	-0.35	-0.76*	-0.40	0.81*	0.41
ECs4555	*espD*	-0.45	0.40	-0.04	-0.36	1.46*	1.10*
ECs4556	*espA*	0.06	0.91*	0.96	0.60	2.01*	2.61*
ECs4557	*sepL*	0.50	0.40	0.90	0.75	2.03*	2.79*
ECs4558	*escD*	0.61	0.19	0.80	0.40	1.48*	1.88*
ECs4559	*eae*	-0.34	0.14	-0.19	-0.16	1.89*	1.73*
ECs4560	*cesT*	-0.65	0.97	0.32	0.37	2.657*	3.03*
ECs4561	*tir*	0.27	0.28	0.55*	-0.10	2.445*	2.34*
bECs4562	*map*	0.69	0.21	0.90*	0.57	1.57*	2.13*
ECs4563	*cesF*	0.89	0.56	1.46	0.57	0.87	1.43*
ECs4564	*espH*	0.79	0.39	1.18*	0.89	2.34*	3.23*
ECs4565	*sepQ*	0.10	0.13	0.23	0.52	2.12*	2.64*
ECs4566	*orf16*	0.08	-0.33	-0.25	1.32	1.78*	3.10*
ECs4567	*orf15*	0.16	-0.32	-0.16	1.18	1.08*	2.26*
ECs4568	*escN*	-0.10	0.13	0.04	0.08	1.51*	1.59*
ECs4569	*escV*	0.04	0.73*	0.77*	-0.02	1.067*	1.04*
ECs4570	*orf12*	-0.03	0.86	0.83	0.25	0.906	1.16*
ECs4571	*sepZ*	0.81	0.82*	1.62*	0.53	1.49*	2.02*
ECs4572	*rorf8*	-0.39	1.30*	0.91	0.07	2.27*	2.34*
ECs4573	*escJ*	-0.70	1.38*	0.68	0.39	2.57*	2.96*
ECs4574	*sepD*	-0.22	1.12*	0.90	0.72	2.39*	3.10*
ECs4575	*escC*	-0.32	1.46*	1.14*	0.55	2.29*	2.84*
ECs4576	*cesD*	0.24	1.33*	1.57*	0.76	2.93*	3.68*
ECs4577	*orf11*	0.43	2.77*	3.19*	1.46	2.21*	3.67*
ECs4578	*grlR*	0.71	2.63*	3.35*	1.22	1.93*	3.14*
ECs4579	*grlA*	0.95	1.72*	2.66*	1.28	0.94	2.22*
ECs4580	*ssaU*	0.27	1.00	1.27	0.49	0.44	0.93*
ECs4581	*escT*	0.92	1.15*	2.07*	0.75	1.2*	1.95*
ECs4582	*escS*	0.08	0.35	0.43	1.46	1.04	2.50*
ECs4583	*escR*	0.32	0.91	1.23	0.33	1.41*	1.74*
ECs4584	*escL*	0.73	0.59	1.31	0.74	0.99*	1.73*
ECs4585	*orf4*	0.47	0.51	0.97	0.75	1.09*	1.84*
ECs4586	*orf3*	0.35	0.21	0.56	1.15	1.14*	2.29*
ECs4587	*orf2*	0.15	0.19	0.34	1.46	1.69*	3.15*
ECs4588	*ler*	0.93	-0.12	0.81	1.20	1.52*	2.71*
ECs4590	*espG*	0.66	-0.03	0.63	0.19	1.65*	1.84*
DEG totals (log_2_FC≥1.5)		0	3	6	0	23	33

*, FDR *P* ≤0.05

1x, 20 μg/L SMX and 4 μg/L TM; 27x, 540 μg/L SMX and 108 μg/L TM

The genes encoding effector proteins secreted via TTSS as identified by Tobe *et al*. [33] were also differentially expressed in a concentration- and time-dependent pattern, similar to the LEE genes (Table 5). The majority of DEGs (≥3-fold difference, false discovery rate (FDR) *P*≤0.05) were identified in the 27vs1 or 27vs0 samples at 12 hours. In general, more DEGs were up regulated than down regulated and the down-regulated DEGs were clustered in Sps 9, 10, and 11. At five hours, only three DEG were identified. ECs0073 and ECs1127 were decreased >3 fold by 27x SMX-TM, but neither was differentially expressed at 12 hours. The only other DEG at five hours (ECs4657) was up-regulated by the 27x SMX-TM concentration at both five hours and 12 hours. Interestingly, ECs0073 was the only down-regulated DEG (significant when using the 3-fold threshold) under any condition that was not located in an Sp or SpLE region. No DEG was observed at either time point in the 1x SMX-TM vs. untreated samples, suggesting that effector protein expression is highly dependent on SOS induction.

**Table 5:** Differential expression (log_2_ FC) of putative EHEC effector genes derived from RNA-Seq comparisons of serotype O157:H7 strain PA20 grown for five hours or 12 hours in LB-NS at 30°C in the presence of different SMX-TM concentrations as shown. Comparisons with a log_2_ FC ≥1.5 or ≤-1.5 (FC≈3) are shown in bold.

Locus tag	Gene	Effector	LTR	1vs0^5h^	27vs1^5h^	27vs0^5h^	1vs0^12h^	27vs1^12h^	27vs0^12h^
ECs0025	hypothetical protein	EspX1	No^$^	0.23	0.67	0.91	1.05	0.30	1.35*
ECs0061	hypothetical protein	EspY1	No^#^	0.03	-0.55	-0.52	0.06	1.12*	1.18*
ECs0073	hypothetical protein	EspY2	No^$^	0.14	-2.16*	-2.02*	-0.07	-0.09	-0.16
ECs0472	hypothetical protein	EspY3	No^#^	0.29	0.30	0.59	0.42	-0.48	-0.06
ECs0846	hypothetical protein	NleB2-1	Sp3	0.16	0.08	0.23	1.03	0.94*	1.96*
ECs0847	hypothetical protein	NleC	Sp3	-0.02	0.37	0.35	0.57	0.42	0.99*
ECs0848	hypothetical protein	NleH1-1	Sp3	0.61	-0.17	0.44	0.42	1.36*	1.78*
ECs0850	hypothetical protein	NleD	Sp3	0.46	-0.85*	-0.39	0.45	0.38	0.82*
ECs0876	hypothetical protein	EspX2	No^$^	-0.08	0.63	0.56	0.27	0.72*	0.98*
ECs1126	EspF-like protein	EspF2-1’	Sp4	-0.09	0.35	0.25	0.35	0.11	0.46
ECs1127	hypothetical protein	EspV’	Sp4	0.79	-1.52*	-0.73	0.03	0.49	0.51
ECs1560	secreted effector protein	EspX7	Sp6	0.04	-0.45	-0.42	0.41	-1.11*	-0.70*
ECs1561	hypothetical protein	EspN	Sp6	0.18	0.56	0.74	1.02	0.21	1.23*
ECs1567	hypothetical protein	EspO1-1	Sp6	0.34	-0.26	0.07	0.43	0.21	0.64
ECs1568	hypothetical protein	EspK	Sp6	0.09	-0.59*	-0.50	0.32	-0.86*	-0.54*
ECs1812	hypothetical protein	NleA	Sp9	0.16	0.66*	0.81*	0.17	-1.71*	-1.54*
ECs1814	hypothetical protein	NleH1-2	Sp9	0.11	-0.87*	-0.77	0.57	-2.15*	-1.58*
ECs1815	hypothetical protein	NleF	Sp9	0.10	-0.59	-0.49	1.05	-2.07*	-1.02*
ECs1821	hypothetical protein	EspO1-2	Sp9	0.74	-0.73	0.01	0.95	0.45	1.4*
ECs1824	hypothetical protein	NleG	Sp9	-0.10	-0.33	-0.43	-0.01	0.62	0.61
ECs1825	BfpT-regulated chaperone-like protein	EspM1	Sp9	0.44	-1.09*	-0.66	0.43	-0.41	0.018
ECs1994	hypothetical protein	NleG2-2	Sp10	0.28	-0.8*	-0.52	0.80	-1.59*	-0.8*
ECs1995	hypothetical protein	NleG6-1	Sp10	0.00	-0.10	-0.10	0.78	-1.21*	-0.43
ECs1996	hypothetical protein	NleG5-1	Sp10	0.40	-0.79*	-0.39	0.46	-0.28	0.18
ECs2073	hypothetical protein	EspR1	No^$^	0.54	0.90*	1.44*	0.99	0	0.99*
ECs2074	hypothetical protein	EspR2’	No^$^	0.18	1.17*	1.35	1.06	0.04	1.1*
ECs2075	IpaH-like protein	EspR2’	No^$^	0.66	-0.40	0.25	0.04	1.48	**1.51**
ECs2154	hypothetical protein	NleG5-2	Sp11	0.13	-0.19	-0.06	0.38	-1.17*	-0.79*
ECs2155	hypothetical protein	NleG6-2	Sp11	0.05	-0.02	0.03	0.69	-1.74*	-1.05*
ECs2156	hypothetical protein	NleG2-3	Sp11	0.43	-0.6*	-0.17	0.70	-1.89*	-1.19*
ECs2226	hypothetical protein	NleG7’	Sp12	0.69	-0.73	-0.04	0.83	3.2*	4.03*
ECs2227	hypothetical protein	NleG3’	Sp12	0.68	-1.40	-0.72	0.31	3.0*	3.28*
ECs2229	hypothetical protein	NleG2-4’	Sp12	0.90	-0.96*	-0.06	0.92	-1.36*	-0.45
ECs2427	hypothetical protein	EspL1	No^#^	0.15	-0.13	0.01	0.09	-0.38	-0.29
ECs2714	hypothetical protein	EspJ	Sp14	0.61	-1.20*	-0.59	1.30	0.24	1.54*
ECs2715	EspF-like protein	TccP	Sp14	-1.22	-0.09	-1.31*	-0.66	0.48	-0.19
ECs3485	chaperone-like protein	EspM2	Sp17	0.15	-0.33	-0.17	0.35	3.88*	4.23*
ECs3486	hypothetical protein	NleG8-2	Sp17	0.02	0.10	0.12	0.30	3.68*	3.98*
ECs3487	hypothetical protein	EspW	Sp17	0.15	0.66	0.81	0.83	2.91*	3.74*
ECs3488	hypothetical protein	NleG6-3’	Sp17	0.28	-0.09	0.19	0.91	-0.21	0.7*
ECs3855	enterotoxin	EspL2	SpLE3	0.08	0.51	0.59	0.58	0.02	0.6
ECs3857	hypothetical protein	NleB1	SpLE3	-0.11	0.35	0.23	0.50	0.21	0.72
ECs3858	hypothetical protein	NleE	SpLE3	-0.07	0.32	0.24	0.26	0.34	0.6
ECs4550	protein EspF	EspF1	SpLE4	-1.34	0.57	-0.77	-0.86	2.82*	1.93*
ECs4554	protein EspB	EspB	SpLE4	-0.41	-0.35	-0.75*	-0.40	0.81*	0.41
ECs4561	hypothetical protein	Tir	SpLE4	0.27	0.28	0.55*	-0.10	2.45*	2.32*
ECs4562	hypothetical protein	Map	SpLE4	0.69	0.21	0.90*	0.57	1.57*	2.11*
ECs4564	hypothetical protein	EspH	SpLE4	0.79	0.39	1.18*	0.89	2.34*	3.23*
ECs4571	SepZ	EspZ	SpLE4	0.81	0.82*	1.62*	0.53	1.49*	2.02*
ECs4590	protein EspG	EspG	SpLE4	0.66	-0.03	0.63	0.19	1.65*	1.84*
ECs4642	hypothetical protein	EspL3’	No^$^	0.72	-0.37	0.36	0.12	0.8*	0.92*
ECs4643	hypothetical protein	EspL3’	No^$^	0.03	0.91*	0.93*	-0.11	1.33*	1.22*
ECs4653	hypothetical protein	EspY4	No^$^	0.56	0.72	1.27*	0.26	0.66	0.92*
ECs4654	hypothetical protein	EspX3’	No^$^	0.91	0.13	1.05	0.60	2.39*	2.98*
ECs4655	hypothetical protein	EspX3’	No^$^	0.24	0.66	0.89	-0.19	4.19*	3.99*
ECs4657	hypothetical protein	EspY5’	No^$^	0.93	0.67	1.60*	0.98	3.07*	4.05*
ECs4935	regulator of acetyl CoA synthetase	EspL4	No^#^	0.17	0.52	0.69	1.02	0.72*	1.73*
ECs5021	hypothetical protein	EspX4	No^#^	-0.34	1.44*	1.1*	0.82	0.9*	1.72*
ECs5048	hypothetical protein	EspX5	No^#^	0.91	-0.04	0.87	0.76	0.87*	1.63*
ECs5295	hypothetical protein	EspX6	No^#^	0.80	-0.53	0.27	0.22	3.15*	3.37*
DEG Totals (log_2_FC ≥1.5; FDR≤0.05)				0/60	2/60	2/60	0/60	20/60	23/60

*, FDR *P* ≤0.05

^$^, O-island as described in Tobe *et al*. [33]

^#^, C-island as described in Tobe *et al*. [33]; 1x, 20 μg/L SMX and 4 μg/L TM; 27x, 540 μg/L SMX and 108 μg/L TM

There was also a strong time effect on *stx* gene expression. Both *stx*_*1*_ and *stx*_*2*_ were differentially expressed only by 27x SMX-TM in parallel with expression of the SOS genes *recA* and *lexA* (Table 2). At five hours, both *stx* genes were significantly reduced compared to controls, but by 12 hours both were strongly increased, with *stx*_*2*_ being as much as 128-fold higher than untreated controls. Thus, both the degree of *stx* expression and the direction (up or down) of the expression were dependent on time.

### Pch regulation of *ler*

Major regulators controlling Ler and LEE are *grlA* and the *perC* homologues (*pch*). The group 1 *pch* (*ABC*) were discussed briefly earlier and showed strong 27x SMX-TM-induced expression at 12 hours (Table 2). In contrast to the group 1 *pch* genes, SMX-TM reduced expression of group 3, *pchE*. The *pchE* reductions did not surpass the 3-fold threshold (-1.5≥ log_2_ FC ≥1.5) but 2-fold decreases were observed for both 27vs0^12h^ and 27vs1^12h^ samples. Therefore, PchE levels are likely reduced by SMX-TM and would not contribute to an additive PerC regulatory effect generated by group 1 Pch members. Group 2 *pchD* was the least affected of the 5 homologues.

The three group 1 *pch* genes differ at just three nucleotide positions in their 315-bp sequences (16, 172, and 183) resulting in a unique amino acid for each member. This lack of sequence diversity suggests that the group 1 *pch* expression reads could have been ambiguously assigned during RNA-Seq read mapping. Therefore, we also used SNP analysis to differentiate expression levels between those three genes (see methods). The log_2_ FC calculated for group 1 members A, B, and C in 27vs0^12h^ were -0.33 (*P* = 0.73), 1.74 (*P* = 0.09), and 2.99 (*P* = 1.54E-06), respectively. The mean 27x SMX-TM reads per kilobase of transcript per million mapped reads (RPKM) ± standard deviation (SD) were 7.66 ± 8.19 (*pchA*), 11.44 ± 7.36 (*pchB*), and 90.73 ± 12.01 (*pchC*). Only *pchC* showed a significant expression change between the 27x SMX-TM treated and untreated samples, that being a nearly 8-fold increase. There was also a >3-fold increase in *pchB* but the mean fell just short of the *P* = 0.05 significance level.

**Fig 2:**
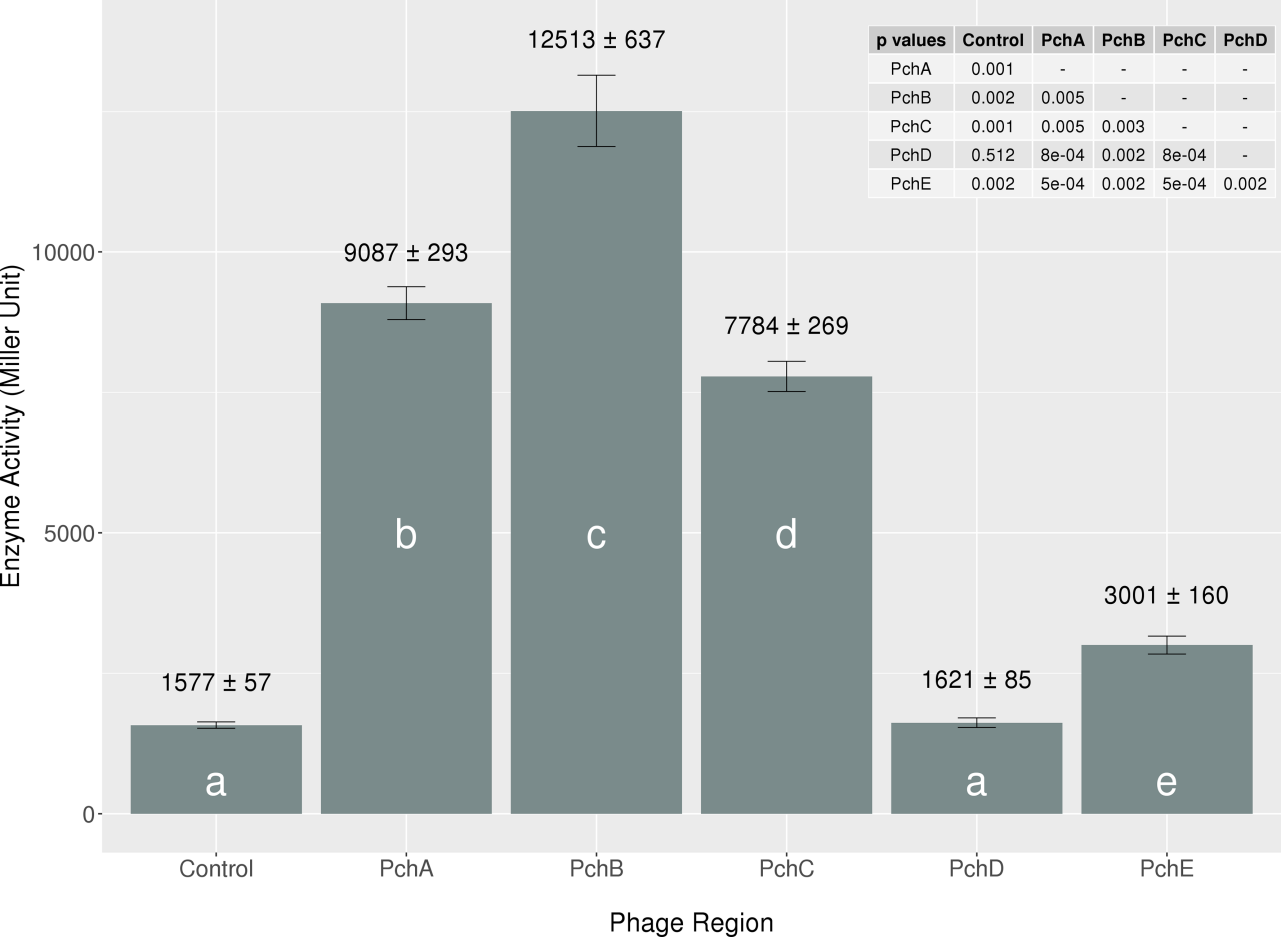
Effect of Pch homologues on *ler* expression. Comparison of the ß-galactosidase activities of strain MG1655Δ*yfdN* containing a *ler*::*lacZ* chromosomal fusion and transformed with plasmid pSE380 or pSE380 carrying cloned *pch* genes as indicated. The enzymatic activity is reported as Miller units. Bars represent the mean of three individual samples tested in lysogeny broth (LB) and induced with 0.1 μM IPTG. Strain means with the same letter are not significantly (*P*≤0.05) different from each other.

Because of the variable expression patterns and the sequence differences among the *pch* genes, we compared the ability of the five different PA20 Pch homologues to directly activate a chromosomal *lacZ*:*ler* fusion constructed in *E*. *coli* MG1655 carrying a deletion of the K12 unique PerC homologue *yfdN* (Fig 2). Recombinant Pch proteins were induced from plasmid pSE380 using 0.1 μM IPTG. Higher IPTG concentrations restricted the growth of the strains bearing *pchB* and *pchD*. All of the three group 1 genes increased (*P*<0.05) *ler* transcription at least 4 fold compared to the pSE380 control (mean enzyme activity in Miller units ± SD = 1,577 ± 56). PchB (12,513 ± 637) induced the highest *ler* activity and PchA (9,087 ± 292) induced more than PchC (7,783 ± 268). In contrast, the effect of PchD and PchE on *ler* transcription was minimal. PchE (3,001 ± 159) resulted in a small (<2-fold) but significant (*P*<0.05) increase in promoter activity while PchD (1,620 ± 85) showed no difference (*P*>0.05) compared to the control carrying only pSE380. Therefore, each of the *pch* group 1 genes was capable of driving *ler* but unambiguous SNP mapping of group I *pch* transcripts indicated that *pchC* was the controlling member following HTR induction because of its 8-fold expression increase.

### Temperature effects are stronger for biofilm than for virulence genes

We also compared SMX-TM induced expression changes at two different temperatures following strain growth on a solid T-agar (Table 6). Using qRT-PCR, the *stx*_*1*_ and LEE genes (*ler*, *eae*, and *tir*) showed small SMX-TM induced expression increases at both 37°C and 30°C, but there was little difference in the magnitude of expression change between temperatures. SMX-TM-induced differential expression of *stx*_*2*_ was greater at 30°C than 37°C (114 ± 29 and 43±10, respectively) but the increases compared to controls were dramatic for both temperatures. Such a finding indicates that the strength and timing of SOS induction were more important than temperature for virulence gene regulation. In contrast, temperature had more profound effects on SMX-TM induction of the biofilm genes. SMX-TM-induced differential expression of central regulator, *csgD*, was markedly greater (>82-fold suppression) at 37°C than at 30°C (>4-fold suppression). Induced expression changes associated with biofilm maturation gene *pgaD* were more modest being >2-fold but the effect switched from activation at 37°C to suppression when tested at 30°C. Although not an objective of this study, a comparison of differential gene expression for cultures grown in liquid versus a solid surface is possible for a limited number of DEGs by comparing the qRT-PCR results (Table 6) with the RNA-Seq data (Tables 2 and 4), respectively. Compared with the earlier RNA-Seq results from LB-NS broth (shown as log_2_ FC), the 30°C results from T-agar (shown as FC) were similar in expression trends and direction. However, the magnitude of expression changes for most all samples was slightly lower on agar surfaces suggesting that either the strength or timing of expression responses may differ between culture broth and agar-solidified media.

**Table 6:** Differential FC expression of selected biofilm and virulence genes of strain PA20 grown for 12 hours on T-agar in the presence of 540 μg/L SMX and 108 μg/L TM (27x concentration) at 30°C and 37°C. RNA from three independent samples were analyzed by qRT-PCR. Standard deviations are shown in parenthesis.

Gene	30°C	37°C
	qRT-PCR	qRT-PCR
*stx1*	2.42 (0.36)	-1.35 (0.35)
*stx2*	114.29 (28.99)	43.47 (10.44)
*ler*	3.26 (0.5)	1.52 (0.1)
*espP*	1.86 (0.32)	-1.5 (0.23)
*eae*	1.47 (0.22)	1.44 (0.38)
*tir*	2.26 (0.34)	2.23 (1.07)
*pgaD*	2.61 (0.52)	-2.39 (1.01)
*csgD*	-4.79 (1.13)	-82.21 (31.20)
distal *mlrA*	62.22 (12.49)	20.66 (7.51)
*recA*	2.93 (0.61)	2.07 (0.4)
*lexA*	1.68 (0.32)	2.66 (0.46)

## Discussion

The primary purpose of this study, conducted using a clinical isolate at 30°C, was to determine whether prophage induction, following SMX-TM exposure, affects biofilm regulators *csgD* or *mlrA*. In addition, we document the affect of SMX-TM on virulence and HTR gene expression at temperatures less than 37°C.

### SMX-TM induction represses rather than enhances *csgD* expression

Like for most clinical O157:H7 strains, PA20 biofilm formation is attenuated by a prophage insertion in *mlrA*. Prophage excision or the expression of distal *mlrA* segments induced by SMX-TM could have restored *csgD* function but instead, *csgD* was further repressed. A few reformed native *mlrA* transcripts without the prophage were identified in some samples but were insufficient for comparative analyses and likely had little effect on *csgD* expression. Distal MlrA fragments, although highly expressed following 27x SMX-TM exposure, also failed to increase *csgD* expression. We also cannot exclude the possibility that the *mlrA* fragments could have contributed to the slight suppression. Past studies have shown that distal MlrA fragments could either increase or decrease CsgD-dependent properties, dependent on temperature and fragment length [38]. The SMX-TM concentrations in this study activated the SOS DNA repair system but clearly did not provide a mechanism to overcome existing barriers in *csgD*-dependent biofilm regulation, leaving clinical strains of serotype O157:H7 at a disadvantage when transitioning to environmental conditions.

### HTR induction by SMX-TM is dependent on concentration and exposure duration

Various studies have documented the damaging effects of certain antibiotics on serotype O157:H7 DNA, leading to initiation of the SOS response and strong induction of virulence genes in the prophage regions. Most of the studies reported gene expression in broth cultures at 37°C following short exposures to a single concentration of the antimicrobial agent. This study tested a lower temperature, compared some gene expression on solid agar, tested two different concentrations of antibiotics, and tested both a short and a long exposure period to encompass more environmental conditions. Our findings were similar to other published reports, but showed greater numbers of DEG. In one microarray study, EDL933 was subjected to a non-inhibitory concentration of norfloxacin for two hours at 37°C in lysogeny broth (LB) [48]. Using a 2-fold expression threshold, 118 up-regulated and 122 down-regulated genes were differentially expressed including just 85 up-regulated prophage-associated genes. The LEE operon was not strongly affected showing only seven down-regulated genes. However, the SOS genes were not strongly affected suggesting that the norfloxacin concentration or the length of exposure may not have strongly induced the SOS response. Landstorfer *et al*. [49] also performed an extensive RNA-Seq study of O157:H7 strain EDL933 (GenBank:NC 002655) following growth under 11 different conditions, including SMX-TM exposure at a final concentration of 2 μg/ml SMX and 0.4 μg/ml TM, that being ~4-times higher than our 27x concentration. The Landstorfer study found that 12 of 41 genes in the LEE pathogenicity island were significantly differentially expressed (-1≥ log_2_ FC ≥1), 11 (*grlR*, Z5111, Z5114, Z5121-Z5126, Z5128, Z5131) up regulated and one (*espB*) down regulated. In the current study, 33 of 41 LEE genes were differentially expressed in the 27vs0^12h^ samples (all increased). Interestingly, our 27vs0^5h^ samples showing six up-regulated genes and significant down-regulation of *espB*, more closely resembled their results. It is unclear how long the samples were exposed to SMX-TM in the Landstorfer study, but the results of the two studies are more similar when comparing the shorter (five hour) exposure time in our study. The effect of temperature on the SMX-TM induction was not a specific variable tested in either study and therefore we cannot speculate on that relationship. However, the duration of SMX-TM exposure alone clearly had dramatic effects on the virulence gene expression in HTR at lower than host temperatures.

### SMX-TM induction of virulence genes is not restricted to host temperatures

There were also some interesting observations regarding temporal regulation of the virulence genes. Induction of the SOS response, as indicated by RecA/LexA increases, was strong at both five hours and 12 hours but expression changes in the HTR were markedly stronger in the 12-hour samples. This disparity could be due to timing differences of specific virulence gene regulators or merely the lag time between SOS induction and the de-repression of lysogeny. With regard to the LEE operon, which is not controlled by promoters associated with the prophage life-cycle, the expression differences between five hours and 12 hours are likely due to timing differences between the expression of *grlAR* and *pch* genes. In strain PA20, the significantly higher expression of *pchC* revealed using an unambiguous SNP analysis suggests that PchC may be the major contributor stimulating *ler* expression.

The regulation of *stx* genes under these conditions is especially noteworthy. Like most virulence factors in the HTR, both *stx*_*1*_ and *stx*_*2*_ showed significant, ≥3-fold expression increases only in the 27x samples at 12 hours. However, unlike most HTR-encoded virulence genes, both were significantly repressed more than 2-fold at five hours. In the study by Herold *et al*. [48] using norfloxacin, *stx*_*2*_ increased more than 100 fold, despite poor induction of the SOS response, but *stx*_*1*_ showed little change. In the Landstorfer study [49] with SMX-TM, there was no significant change with either *stx*_*1*_ or *stx*_*2*_. Understanding the mechanism of the *stx* repression in our 5-hour samples in the presence of strong SOS induction may provide clues for new strategies to reduce Stx in clinical settings.

From a biological perspective, increasing virulence gene expression under environmental conditions seems energetically wasteful; but, considering the strong expression of genes associated with the SOS response in our 27x SMX-TM comparisons, increased expression of HTR-associated virulence genes is not surprising. Prophage induction is an obvious driving force for virulence gene expression as has been shown for *stx*_*2*_ and the LEE virulence genes [16, 50]. It is not clear if virulence gene expression in environmental settings, as shown here, serves a specific purpose or if it merely reflects inefficient control in the prophage elements that–from an evolutionary perspective–are relatively new acquisitions in the *E*. *coli* genome. Insects [51] or earthworms [52] may serve as vectors for the persistence of STEC in the environment or for the movement of STEC to or between food plants or animals. While the present study demonstrates the potential for expression of virulence factors under environmental conditions in antibiotic stressed cells of *E*. *coli* O157:H7, little has been reported on the potential virulence of STEC in either insects or earthworms. However, a study in a silkworm moth model showed that virulence determinants *stx*_*1*_, *stx*_*2*_ and *eae* did not effect serotype O157:H7 viability in that insect; albeit, testing was conducted at mammalian host temperature, 37°C [53]. Clearly, more studies are needed to define absolute expression levels of the phage-encoded proteins and their specific roles at different temperatures.

In this study, we found that high sub-lethal SMX-TM concentrations initiated the SOS response early but strong induction in HTR, including most virulence genes, was delayed until a later time-point. Neither high or low levels of SMX-TM stimulated *csgD* induction at either time point, but both levels resulted in slight repression. Therefore, application of antimicrobial agents that initiate the SOS response would likely have little effect on biofilm formation in those O157:H7 strains carrying a prophage in *mlrA*. We also found that full activation of Ler-dependent PA20 genes is controlled by *grlA* together with the combined, but unequal, contributions of *pch* homologues A, B, and C in a two-step, time-dependent process. Finally, we found that *stx*_*2*_ expression, which is strongly dependent on prophage induction, was greatly enhanced at 12 hours but suppressed during early SOS initiation by the higher SMX-TM concentration.

## Materials and methods

### Strains, growth conditions, and molecular biology techniques

*E*. *coli* serotype O157:H7 PA20 is a Stx1+/Stx2+ clinical isolate from the PA Department of Health that has been studied extensively in our laboratory [35, 38, 45]. The entire genome sequence of strain PA20 was recently reported, deposited in GenBank [GenBank Accession # CP017669 (genome) and CP017670 (plasmid)] [46], and showed strong similarity to strain Sakai. NEB 5-alpha (New England Biolabs, Inc) and One Shot PIR1 (Invitrogen) were used as *E*. *coli* host strains for intermediary cloning steps. PA20 cultures for RNA-Seq were grown in LB-NS with and without added SMX-TM at designated levels for the indicated times and temperatures. Working stocks were tested and maintained using LB (Miller formulation) or LB agar. Plasmid pSE380 derivatives were induced by 0.1 μM IPTG. Higher IPTG concentrations restricted the growth of the strains bearing *pchB* and *pchD*.

Chromosomal DNA was isolated using the DNeasy Blood and Tissue Kit (Qiagen) or Qiagen Genomic-tip 100/G (Qiagen). Primers are listed in S1 Table. The Qiagen multiplex PCR kit was used for routine PCR amplifications (Qiagen).

### Construction of recombinant plasmids and bacterial strains

A DNA fragment containing 655 bp of the *ler* promoter and the first 22 *ler* codons was PCR amplified from strain PA20 using primers LERLacF/LERLacR, cloned into the *Sma*I/*Bam*HI sites of plasmid pMCI002, and swapped with the *lacZ* promoter in strain MG1655 as previously described to produce strain MG1655LacLer (S1 Table) [54]. The MG1655LacLer *yfdN* gene was replaced by a neomycin cassette using RedET recombination and primers yfdNred50F/yfdNred50R to produce MG1655LL-yfdN as previously described (Genebridges GmbH; S1 Table) [55]. Each of the (5) O157:H7 PerC homologues were amplified from PA20 and cloned into the *Nco*I/*Hin*dIII or *Eco*RI/*Hin*dIII site of plasmid pSE380 to produce p380PchA, p380PchB, p380PchC, p380PchD, and p380PchE using primers listed in S1 Table.

### RNA isolation and cDNA preparation

RNA for qRT-PCR analyses was harvested from strains growing on T-agar surfaces [56]. Five ml of LB were inoculated from glycerol stocks of strain PA20 and grown for 16 hours at 37°C. A 1-ml aliquot of each was centrifuged at 13,000 xg for 2.5 min., re-suspended in 100 μl LB, spin-plated on 30°C-pre-warmed T-medium agar with or without SMX-TM as designated, and incubated 12 hours at 30°C or 37°C. Total cells from each plate were collected using a sterile cotton swab (Puritan Medical Products Company, LLC) and suspended in RNAzol RT (Molecular Research Center). For RNA-Seq studies, strains were grown in broth to facilitate processing greater sample numbers. Sixty μl of each 16-hour culture was inoculated into 20 ml LB-NS broth and incubated at 30°C. Samples of 5 ml at 5 hours and 10 ml at 12 hours were harvested by centrifugation and suspended in RNAzol RT. Total RNA was isolated following the manufacturer’s protocol (including phase separation with 4-bromoanisole) and re-suspended in 40 μl nuclease-free water. Contaminating DNA was removed from RNA samples by DNase I digestion using the TURBO DNA-free kit (Ambion). Three samples for each condition (antimicrobial concentration and time point) were processed for screening by RNA-Seq. cDNA from 1 μg total RNA, collected from three separate agar plates for each tested sample, was generated using the High Capacity cDNA Reverse Transcriptase Kit (ThermoFisher Scientific).

### RNA-Seq

RNA-Seq was performed by ProteinCT Biotechnologies, LLC (Madison, WI) and expression analysis was performed by BioInfoRx, Inc. (Madison, WI) as previously described [57]. Briefly, total RNA was treated with RiboZero kits for bacteria (Epicentre) to reduce bacterial rRNA, followed by library preparation with the Illumina TruSeq strand specific mRNA sample preparation system (Illumina). After RNA fragmentation, strand specific libraries were constructed by first-strand cDNA synthesis using random primers, sample cleanup and second-strand synthesis using DNA Polymerase I and RNase H. A single 'A' base was added to the cDNA fragments followed by ligation of the adapters. Final cDNA libraries were generated by further purification and enrichment with PCR, followed by a quality check using a Bioanalyzer 2100 (Agilent). The libraries were sequenced (1x50bp) using the Illumina HiSeq2500, with a final count of ~15 million or more reads per sample.

### RNA-Seq data analysis

RNA-Seq data were mapped to the *E*. *coli* O157:H7 Sakai genome using the Subjunc aligner program from the Subread package (v1.4.6) (http://bioinf.wehi.edu.au/subread/) [58]. The alignment Bam files were compared against the gene annotation general feature format (GFF) file, and raw counts for each gene were generated using the featureCounts tool from Subread. The raw counts data of the expressed genes was normalized for RNA composition using the trimmed mean of M values (TMM) method (http://www.ncbi.nlm.nih.gov/pubmed/20196867) from the Empirical analysis of Digital Gene Expression Data in R (EdgeR) package (https://bioconductor.org/packages/release/bioc/html/edgeR.html), then transformed to log2CPM (counts per million) values using the voom method (http://www.ncbi.nlm.nih.gov/pubmed/24485249) from the R LIMMA package (https://bioconductor.org/packages/release/bioc/html/limma.html) [59]. Next, a linear model was built for each comparison using the R LIMMA package and statistics for differential expression analysis were computed. False discovery rates (FDR) were determined and used to reduce false positive results that can occur when performing multiple comparisons. To filter for differential expression, two-fold or three-fold change with a FDR ≤0.05 were used as the threshold. Functional annotation was done using the Database for Annotation, Visualization and Integrated Discovery (DAVID) Bioinformatics Resources 6.7, NIAID, NIH (http://david.abcc.ncifcrf.gov). Although the genome sequence of strain PA20 has been deposited with GenBank, the Sakai annotation is more complete than the automated PA20 version. Therefore, gene comparisons were described using the Sakai locus-tag designations.

The three group 1 *pch* genes (*ABC*) have identical sequence in their coding regions except for variations at positions 16, 172 and 183, which give each member a unique sequence identity. The raw reads were mapped to *pchA* to get the total read count for the three genes and the reads that spanned the three unique positions were used to determine the percentage for each gene. The final expression level for each *pch* gene was computed from the total read count and individual percentages, and were used to estimate the fold change difference for each *pch* between 27x SMX-TM-treated and untreated samples at 12 hours.

### qRT-PCR

qRT-PCR was performed on a 7500 Fast Real Time PCR System (Applied Biosystems) using 20-μl reactions containing 20 ng cDNA (or 20 ng DNase-treated RNA as a negative control), 10 μl Fast SYBR Green Master Mix (Applied Biosystems), and each primer at a final concentration of 0.5 μM (Integrated DNA Technologies). The *gyrA* gene was used as a reference to normalize the results. Primers used are shown in S1 Table. qRT-PCR data were analyzed using the FC = 2-ΔΔCT method [60]. The mean log_2_ FC for three trials of qRT-PCR for the selected genes along with SD are reported and compared with corresponding RNA-Seq results.

### β-galactosidase assays

Overnight 18-hour LB cultures of tested strains were diluted 1:100 in fresh LB broth, grown one hour, induced with IPTG at a final concentration of 0.1 μM for two hours, and 100 μl was assayed for β-galactosidase activity by the Miller protocol [61]. The results of three independent samples were analyzed in R [62]. Strain variance was assessed with the Bartlett’s test and differences in enzyme activities were determined using the nonparametric Kruskal-Wallis test [63, 64]. Multiple pairwise t-test comparisons were performed to determine the statistical differences in enzyme activity between the grouped strains. The t-tests were performed without the assumption of equal variance (pairwise.t.test option pool.SD = F) and the resulting *P*-values were adjusted with the Benjamini-Hochberg procedure [65].

## Supporting information

S1 Table Primers used in this study. (DOCX)

S2 Table Comparative analyses of RNA-seq data. The RNA-seq data were compared to determine the log_2_ fold change (Log_2_FC) in gene expression and associated false discovery rates (FDR) for different antibiotic concentrations [27x, 1x and no antibiotic (0x)] at both five hours and 12 hours (1x antibiotic was 20 ug/L sulfamethoxazole and 4 ug/L trimethoprim). Horizontally transferred regions [prophage regions 1–18, phage-like elements (SpLE1-6), pO157 genes, and pathogenicity islands (EPAI1-6 and LEE)] are noted in the right columns. Sheet 1: The complete set of Log_2_FC and FDR for pairwise comparison of the different antibiotic concentration comparison at both five hours and 12 hours.
For Sheets 2–13 differentially expressed genes (DEG) with a 3-fold or greater differential expression [-1.5≥ log_2_ FC ≥1.5] and a FDR ≤0.05 were considered significant (Sig) and are highlighted yellow (decreased expression) or green (increased expression).
Sheet 2 (1vs0^5h^): Comparison of RNA-seq expression levels of 1x antibiotic to no antibiotic (0x) after five hours of incubation.
Sheet 3 [1vs0^5h^ (1.5Log_2_ Sig)]: The list of DEGs from Sheet 2. Genes with decreased expression are listed followed by genes with increased expression.
Sheet 4 (27vs0^5h^): Comparison of RNA-seq expression levels of 27x antibiotic to no antibiotic (0x) after five hours of incubation.
Sheet 5 [27vs0^5h^ (1.5Log_2_ Sig)]: The list of DEGs from Sheet 4. Genes with decreased expression are listed followed by genes with increased expression.
Sheet 6 (27vs1^5h^): Comparison of RNA-seq expression levels of 27x antibiotic to 1x antibiotic after five hours of incubation.
Sheet 7 [27vs1^5h^ (1.5Log_2_ Sig)]: The list of DEGs from Sheet 6. Genes with decreased expression are listed followed by genes with increased expression.
Sheet 8 (1vs0^12h^): Comparison of RNA-seq expression levels of 1x antibiotic to no antibiotic (0x) after 12 hours of incubation.
Sheet 9 [1vs0^12h^ (1.5Log_2_ Sig)]: The list of DEGs from Sheet 8. Genes with decreased expression are listed followed by genes with increased expression.
Sheet 10 (27vs0^12h^): Comparison of RNA-seq expression levels of 27x antibiotic to no antibiotic (0x) after 12 hours of incubation.
Sheet 11 [27vs0^12h^ (1.5Log_2_ Sig)]: The list of DEGs from Sheet 10. Genes with decreased expression are listed followed by genes with increased expression.
Sheet 12 (27vs1^12h^): Comparison of RNA-seq expression levels of 27x antibiotic to 1x antibiotic after 12 hours of incubation.
Sheet 13 [27vs1^12h^ (1.5Log_2_ Sig)]: The list of DEGs from Sheet 12. Genes with decreased expression are listed followed by genes with increased expression.
(XLSX)
