# Supplementary material for: Sulfamethoxazole – Trimethoprim represses csgD but maintains virulence genes at 30°C in a clinical Escherichia coli O157:H7 isolate
Source: PLoS One. 2018 May 2;13(5):e0196271. doi: 10.1371/journal.pone.0196271 (PMC5931665; doi:10.1371/journal.pone.0196271)
Supplement: S1 Table — (DOCX) [file pone.0196271.s001.docx]

**Supporting Information**

**S1 Table.** Primers used in this study

| **Primer** | **Sequence** |
| --- | --- |
| 1091PerC380F | aagccatggataatcggaggtcacttatgctacatgatcacg |
| 2182PerC380F | aagccatggataaacggaggttacttatgctacatgatcacc |
| 2737­PerC380F | aagccatggataatcggaggtcacttatgctacatgatcacc |
| PerC380R | aacgagctcttagcatttttttgaccgcgcgtttc |
| 1388PerC380F | aagccatggaaagagtaccccggaagttgtg |
| 1388PerC380R | aacgagctcctgtttgccattccttgcttcatc |
| 1588_PerC380F | aagccatggtgacggtattccgttctgag |
| 1588_PerC380R | aacgagctccggctgttgattagctgtctgg |
| yfdNred50F | acaatgtggactacagccagatcccggcaggattcagggggtgagcatgaattaaccctcactaaagggcg |
| yfdNred50R | ttgtttgttccgcagctccaccagcgcctggcaatatttattactcattataatacgactcactatagggctc |
| lerlacF | aaggaattcgccgaatggatatggacaata |
| lerlacR | aagggatccagctgaatgtatggacttgttg |
| LerProF | aaggaattcactaacgcggttactgttcagc |
| LerProR | aagggatcccccatgctttaatattttaagctattagcg |
| RT-stx1F | attcgctctgcaataggtactcc |
| RT-stx1R | ctatccctctgacatcaactgc |
| RT-stx2F | ccacatcggtgtctgttattaac |
| RT-stx2R | cggtagaaagtatttgttgccgt |
| RT-lerF | agtatatcccagctcttgtaagg |
| RT-lerR | gcaaattgcagttctacagcagg |
| RT-espPF | gtaccatttcctgacttctcacc |
| RT-espPR | gttttgcgtcgcaactgagtgg |
| RT-eaeF | tatcggcgttatccgctttagc |
| RT-eaeR | cctatgaccgtaatggcaatagc |
| RT-tirF | aatcaatgcgccactaagaccg |
| RT-tirR | agagcaggctaaagcagcagg |
| RT-pgaDF | tctgctgacgggttattactgg |
| RT-pgaDR | tcttgcggcgtatattggtagg |
| RT-csgDF | tggacgatatctcttcaggctc |
| RT-csgDR | cgcggtacgggtaatcttcag |
| RT-mrlAF | caaacgctggatcgacaacgg |
| RT-mrlAR | ttgccgctttgcaggtaggtc |
| RT-recAF | agcgtgaaggtaaaacctgtgc |
| RT-recAR | gtcacagatttccagtgcctgc |
| RT-lexAF | gaagaacatctgaaggcgctgg |
| RT-lexAR | aatatgctgttgcgccagaagc |
| SpLE4-F | cccgcacatatgcaagtccagt |
| SpLE4-R | cggttttcactttaacatcggttagc |
| RT-Sp11F | aaccatgccccataacgaagc |
| RT-Sp11R | cgagtaactgaaaggccatcg |
